# Association of Different Host Diets with the Nutritional Composition of the Fall Webworm, *Hyphantria cunea* Drury (Lepidoptera: Erebidae)

**DOI:** 10.3390/insects17060590

**Published:** 2026-06-04

**Authors:** Qiuyu Xu, Kexin Gu, Yanxin Bai, Qun Li, Yanqun Liu

**Affiliations:** Department of Sericulture, College of Bioscience and Biotechnology, Shenyang Agricultural University, Shenyang 110866, China

**Keywords:** *Hyphantria cunea*, proximate composition, minerals, free amino acids, lipids

## Abstract

Different host plants influence the nutritional and metabolic profiles of the fall webworm (*Hyphantria cunea*), a globally invasive pest. This study investigated the proximate composition, free amino acid and lipid profiles of its pupae from larvae reared on three distinct diets (*Robinia pseudoacacia*, *Morus alba* and *Armeniaca sibirica*). The results showed that pupal weight and size varied significantly with host plant. The pupae contained 64.94 ± 1.57% crude protein and 30.82 ± 0.53% crude fat (dry mass basis), with high levels of magnesium and calcium. A total of 82 free amino acids and their metabolites were identified, including all essential and non-essential amino acids. In addition, 1026 lipids were detected, with triacylglycerols as the predominant class. Notably, the levels of linoleic and α-linolenic acid were highest in the pupae fed on *R. pseudoacacia*. Among the three diet groups, the lipid species TG (16:0_16:2_20:5) was identified as a characteristic differential metabolite. These findings show that host-plant-induced nutritional variation reflects metabolic plasticity, which underlies the dietary adaptability and invasion success of this polyphagous pest and also suggestsits potential use as a feed ingredient.

## Simple Summary

We analyzed the weight, size and nutritional composition of fall webworm pupae from larvae reared on three different host plants. The weight and size of the pupae varied significantly with host plant. The pupae were rich in protein, fat, essential amino acids, and diverse lipids. Notably, the nutritional profiles varied with host plant, reflecting the metabolic plasticity of this polyphagous pest. This study not only supports the potential use of *Hyphantria cunea* pupae as a feed ingredient, but also provides insights into how host plants influence the nutritional phenotype of a polyphagous insect.

## 1. Introduction

The establishment and spread of polyphagous insect herbivores outside their native ranges represents a major driver of global ecological change and agricultural loss [1,2]. Among the many traits that facilitate invasion success, dietary breadth—the ability to consume and thrive on a wide range of host plants—is particularly important [3]. However, not all polyphagous species become successful invaders, the key lies in their nutritional plasticity and metabolic adaptability. Polyphagous insects can regulate their intake of key macronutrients, such as protein and carbohydrates. This ability helps them maintain fitness across diverse host plants [4]. Metabolic adaptability encompasses the underlying biochemical adjustments, such as shifts in amino acid and fatty acid metabolism, that allow an insect to maintain fitness on different diets [5]. Understanding how these physiological mechanisms operate in polyphagous pests is therefore fundamental to predicting and managing biological invasions [1].

In addition to their ecological importance, polyphagous insects often accumulate substantial biomass with high levels of protein, essential amino acids, and unsaturated fatty acids [6]. This nutritional richness has drawn growing interest in using insects as alternative protein sources for food and animal feed, particularly in the context of global protein demand and environmental sustainability [7]. For example, under EU Regulation 2015/2283, the European Food Safety Authority (EFSA) has approved dried larvae of *Tenebrio molitor*, *Locusta migratoria*, and *Acheta domesticus* as novel food [8,9,10]. Moreover, insects offer a promising alternative protein source for animal feed, with superior feed conversion ratios as low as 2.0, thus helping to alleviate future global protein shortages [11,12].

*Hyphantria cunea* Drury (Lepidoptera: Erebidae), commonly known as the fall webworm, is native to North America and has invaded Europe and Asia since the 1940s, becoming a major forest pest in these regions [13]. Its larvae are highly polyphagous, feeding on over 600 host plant species spanning trees, shrubs, and crops [13]. In China, it damages more than 300 plant species across 108 genera and 49 families, showing a distinct preference for broad-leaved trees [14]. This pest is recognizable by the characteristic webbed nests it forms on branches during early instars, which often detach in later larval stages. Its strong ecological adaptability, including a broad host range, high fecundity, and wide habitat tolerance, has facilitated its invasive success [15]. To date, research on *H. cunea* has centered largely on its biology, ecological impact, and pest management.

While the fall webworm is known for its polyphagous nature and exhibits clear feeding preferences among host plants, little is known about how these dietary differences affect its metabolic composition. It was hypothesized that host plant identity significantly alters the nutritional and metabolic profiles of *H. cunea* pupae, and that these diet-induced variations reflect the pest’s metabolic plasticity in response to different host plants. To test this hypothesis and to assess the nutritional value of *H. cunea* pupae as a potential feed ingredient, larvae were reared on three host plants, *Robinia pseudoacacia*, *Morus alba* and *Armeniaca sibirica*. A previous study showed that *H. cunea* larvae exhibit significantly different feeding rates among them. Specifically, the feeding rate was highest on *R. pseudoacacia*, lowest on *A. sibirica*, and intermediate on *M. alba* [16]. In this study, the pupae from larvae reared on separately on each ofthose three host plants were analyzed for crude protein, crude fat, minerals, amino acids and their metabolites, and lipid contents. By providing foundational data on compositional variation across different host plants, this work offers insight into the feasibility and consistency of *H. cunea* pupae as a sustainable nutrient source for animal consumption. Meanwhile, elucidating how host plant variation shapes the pupal metabolome not only informs the nutritional consistency of this potential feed resource but also provides a physiological perspective on the dietary adaptability of this polyphagous pest.

## 2. Materials and Methods

### 2.1. Construction of Mapping Population

At the end of June 2022, first and second instar larvae of fall webworm were collected from the tree of *Acer saccharum* in Shenyang Agricultural University (SYAU), Shenyang China (41°48′ N, 123°25′ E). Larvae from three independent nests were collected. From each nest, larvae were randomly divided into the three dietary treatment groups, with at least 150 healthy larvae per group per nest. These groups were then fed on fresh leaves of *R. pseudoacacia*, *M. alba* and *A. sibirica*, respectively, all collected from SYAU. The insects were maintained under laboratory conditions (relative average humidity: 60%, average air temperature: 28 °C) with natural photoperiods (approximately 14 h light: 10 h dark). The insects were kept in a 30-mesh nylon bag (30 × 60 cm). Fresh and healthy leaves were fed every day, and feces and remaining leaves were removed every day. About 5 weeks later, the first pupa appeared. Fresh pupae were collected daily. Each pupa was weighed, and its length and width were measured using a digital caliper (accuracy: 0.01 mm). Length was defined as the maximum distance from the anterior tip of the head to the posterior end of the abdomen. Width was measured at the widest part of the thorax (the broadest transverse section of the pupal body). The pupae obtained from the larvae fed on *R. pseudoacacia*, *M. alba* and *A. sibirica* were designated as Rps, Mal, and Asi pupae, respectively. For each of the three groups, at least 43 pupae were weighed and measured. Some of the pupae were dried at 60 °C to a constant weight for proximate analysis, and some of the pupae were stored at −80 °C after being frozen in liquid nitrogen for amino acid and lipid analysis.

All of the standards of amino acids and lipids were purchased from Sigma-Aldrich (St. Louis, MO, USA) and Avanti Polar Lipids (Alabaster, AL, USA).

### 2.2. Proximate and Minerals Analysis

Fresh samples were dried to constant weight in an air circulating oven (PH070A, Yiheng, Shanghai, China) at 60–70 °C. Crude protein (CP) content was determined by the Kjeldahl method as outlined in the China National Standard (GB/T 6432-2018) [17]. Crude fat (CF) content was measured following the methodology specified in the China National Standard (GB/T 14772-2008) [18]. Mineral content was assessed by using an inductively coupled plasma mass spectrometry method (DB35/T 1142-2020) [19]. All determinations were performed using three biological replicates. Each biological replicate consisted of a group of at least 30 pupae.

### 2.3. Free Amino Acid Analysis

#### 2.3.1. Sample Preparation and Extraction

For each dietary treatment group, five biological replicates were prepared, with four frozen pupae per replicate. After the samples were thawed and smashed, an amount of 0.05 g of the sample was mixed with 500 μL of 70% methanol/water. The sample was vortexed for 3 min (2500 r/min) and centrifuged for 10 min (12,000 r/min, 4 °C). An aliquot of 300 μL of supernatant was transferred into a new centrifuge tube and incubated at −20 °C for 30 min. After incubation, the supernatant was centrifuged at 12,000 r/min for 10 min at 4 °C. Then, 200 μL of resulting supernatant was passed through a Protein Precipitation Plate to LC-MS analysis.

#### 2.3.2. UPLC and ESI-MS/MS

HPLC analysis was performed using an ACQUITY BEH Amide column (2.1 × 100 mm, 1.7 μM, Waters Corporation, Milford, MA, USA). The mobile phase consisted of solvent A (water with 2 mM ammonium acetate and 0.04% formic acid) and solvent B (acetonitrile with 2 mM ammonium acetate and 0.04% formic acid). The gradient program was as follows: 90% B from 0 to 1.2 min, decreased to 60% B at 9 min, then to 40% B from 10 to 11 min, and finally ramped back to 90% B from 11.01 to 15 min. The flow rate was 0.4 mL/min, the column temperature was 40 °C, and the injection volume was 2 μL.

The ESI source operation parameters were as follows: ion source, turbo spray; source temperature 550 °C; ion spray voltage (IS) 5500 V (Positive), −4500 V (Negative); curtain gas (CUR) was set at 35.0 psi; declustering potential (DP) and collision energy (CE) for individual multiple reaction monitoring (MRM) transitions was done with further DP and CE optimization. A specific set of MRM transitions were monitored for each period according to the amino acid eluted within this period. The quantification of amino acid metabolites was achieved using calibration curves for 94 standards (Table S1).

#### 2.3.3. Quantitative Determination of Amino Acid Metabolites

The content of amino acid metabolites was calculated as follows:

The content of amino acid metabolites (ng/g, wet mass) = cV/1000/m

c (ng/mL): concentration, obtained by the ratio of the integral peak area of the sample to the standard curves.

V (µL): extraction solution volume;

m (g): sample weight.

### 2.4. Liquid and Metabolite Analysis

#### 2.4.1. Sample Preparation and Extraction

For each dietary treatment group, five biological replicates were prepared, with four frozen pupae per replicate being sampled. After the samples were thawed and smashed, an amount of 20 mg sample was taken and homogenized in 1mL mixture (methanol, MTBE and internal standard) and steel ball. Take out the steel ball and whirl the mixture for 15 min. Add 200 μL of water and whirl the mixture for 1 min, and then centrifuge it for 10 min (12,000 r/min, 4 °C). The supernatant was concentrated, and then the powder was dissolve with 200 μL reconstituted solution and stored at −80 °C for LC-ESI-MS/MS analysis.

#### 2.4.2. Lipidomic Data Acquisition by LC-ESI-MS/MS

The data-acquistion system comprised an UPLC (Ultra Performance Liquid Chromatography, UPLC) (ExionLC^TM^AD, Sciex, Framingham, MA, USA, https://sciex.com.cn/) (accessed on 25 August 2022) coupled with an MS/MS (Tandem Mass Spectrometry, MS/MS) (QTRAP^®^6500+, Sciex, Framingham, MA, USA, https://sciex.com.cn/) (accessed on 25 August 2022).

UPLC analysis was performed on a Thermo Accucore^TM^C30 column (2.6 μm, 2.1 mm × 100 mm i.d., Thermo Fisher Scientific Inc., Waltham, MA, USA). The mobile phase consisted of solvent A (acetonitrile/water, 60:40 V/V, containing 0.1% formic acid and 10 mmol/L ammonium formate) and solvent B (acetonitrile/isopropanol, 10:90 V/V, containing 0.1% formic acid and 10 mmol/L ammonium formate). The gradient program was as follows: A/B (80:20, V/V) at 0 min, 70:30 at 2.0 min, 40:60 at 4 min, 15:85 at 9 min, 10:90 at 14 min, 5:95 at 15.5 min, 5:95 at 17.3 min, then returned to 80:20 at 17.3 min and held until 20 min. The flow rate was 0.35 mL/min, the column temperature was 45 °C; and the injection volume was 2 μL.

For ESI-MS/MS, the ion source temperature was set to 500 °C. The positive ion spray voltage was 5500 V, and the negative ion spray voltage was −4500 V. Gas settings were as follows: ion source gas 1 at 45 psi, gas 2 at 55 psi, and curtain gas at 35 psi. The declustering potential (DP) and collision energy (CE) were optimized individually for each multiple reaction monitoring (MRM) transition.

#### 2.4.3. Qualitative and Quantitative Analysis of Lipids

Lipid identification was achieved on the basis of the acquired lipid data, including retention time, accurate precursor ions/product ion information, and MS/MS spectrum patterns. Analyst 1.6.3 software (Sciex, Framingham, MA, USA) was performed to compare and match the data with a self-compiled Met Ware database (MetWare Biological Science and Technology Co., Ltd., Wuhan, China, http://www.metware.cn/) (accessed on 25 August 2022). The content of each lipid was quantitatively analyzed using the internal standard method (Table S2). The content of lipid compounds (X, in μg/g) was calculated according to the following formula: X = 0.000001 × R × c × F × V × M/m (X: Lipid compound content, μg/g; R: Ration of the chromatographic peak area of the target compound to the chromatographic peak area of the internal standard; c: Internal standard concentration, μmol/L; F: Internal standard correction factor; V: Volume of the sample extract, μL; M: Precise mass; m: Sample quality, g).

### 2.5. Data Analysis

Statistical analyses were performed using IBM SPSS Statistics version 17.0 (IBM Corp., Armonk, NY, USA). Levene’s test was used to assess the homogeneity of variances across the three dietary groups (Rps, Mal, and Asi). For variables with equal variances, a one-way analysis of variance (ANOVA) followed by Tukey’s honest significant difference (HSD) post hoc test was performed. For variables with unequal variances, Welch’s one-way ANOVA followed by Games -Howell post hoc tests were applied. Statistical significance was set at α = 0.05. Differential free amino acid and lipid identification among the three groups were conducted through Principal Component Analysis (PCA) and Orthogonal Partial Least Squares Discriminant Analysis (OPLS-DA) through SMICA software (Version 14.1).

## 3. Results

### 3.1. Traits and Proximate Composition of H. cunea Pupae

**Figure 1:**
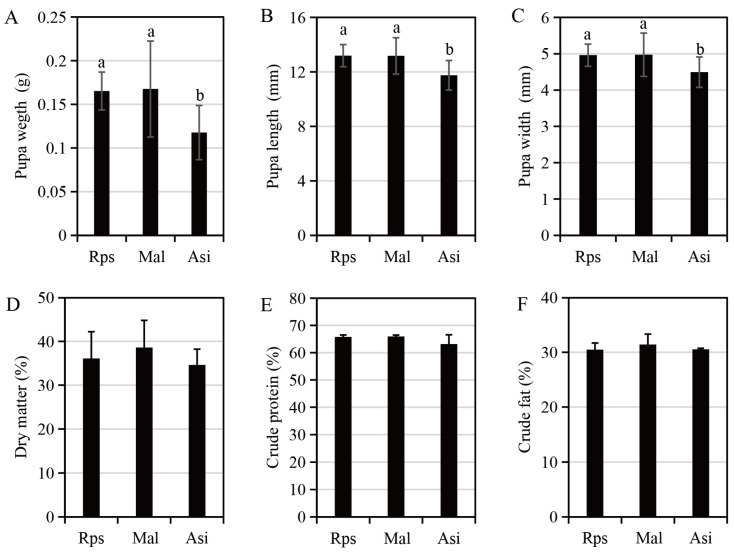
Traits and proximate composition of *H. cunea* pupae. (**A**) pupa weight, (**B**) pupa length, (**C**) pupa width, (**D**) dry matter, (**E**) crude protein, (**F**) crude fat. Data are shown as the mean ± SD (n = 3 biological replicates per group), ANOVA followed by HSD post hoc test was used for multiple comparisons; different lowercase letters indicate significant differences at *p* < 0.05. Rps, Mal, and Asi represent the pupae of larvae reared on leaves of *R. pseudoacacia*, *M. alba* and *A. sibirica*, respectively.

The morphological characters and proximate composition of the fall webworm pupae depend on the host plant. The lowest of weight (0.12 ± 0.03 g) and smallest size (11.75 ± 1.00 × 4.49 ± 0.40 mm) were found in the Asi group, and the highest weight (0.17 ± 0.02 g) and largest size (13.19 ± 0.81 × 4.96 ± 0.31 mm) were found in the Rps group (Figure 1A–C). The dry matter content did not differ significantly among groups and ranged from 34.60 to 38.58% (Figure 1D). The CP and CF contents were 64.94 ± 1.57% dry mass (d.m.) and 30.82 ± 0.53% d.m. on average, and differences among the diets were also insignificant (Figure 1E,F).

### 3.2. Mineral Composition of H. cunea Pupae

**Figure 2:**
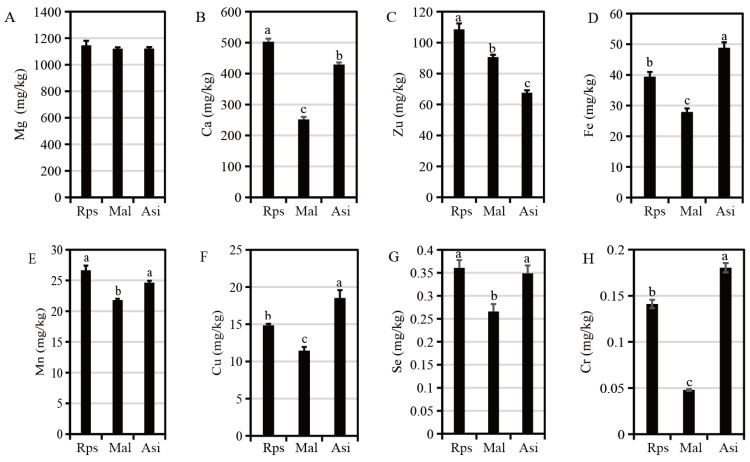
Mineral content of *H. cunea* pupae. (**A**–**H**) The content of Mg, Ca, Zn, Fe, Mn, Cu, Se, and Cr (mg/kg, dry mass). Data are shown as the mean ± SD (n = 3), ANOVA followed by HSD post hoc test was used for multiple comparisons; different lowercase letters indicate significant differences at *p* < 0.05. Rps, Mal, and Asi represent the pupae of larvae reared on leaves of *R. pseudoacacia*, *M. alba* and *A. sibirica*, respectively.

Eight mineral elements were detected in the *H. cunea* pupae. Magnesium (1120.52 ± 1.00–1145.36 ± 34.12 mg/kg d.m.) was the most abundant, followed by calcium (251.84 ± 8.60–502.85 ± 9.78 mg/kg d.m.), zinc (67.58 ± 1.63–108.54 ± 3.87 mg/kg d.m.), iron (27.89 ± 1.11–48.83 ± 1.81 mg/kg d.m.), manganese (21.79 ± 0.20–26.65 ± 0.77 mg/kg d.m.), copper (11.45 ± 0.47–18.54 ±1.04 mg/kg d.m.), selenium (0.27 ± 0.02–0.36 ± 0.02 mg/kg d.m.), and chromium (0.05 ± 0.00–0.18 ± 0.01 mg/kg d.m.). The mineral content varied considerably among pupae from the three groups (Figure 2). Specifically, the Rps group had the highest content of magnesium, calcium, zinc, and manganese (Figure 2A,B,D,E).

### 3.3. Free Amino Acids Profile of H. cunea Pupae

To understand amino acid metabolism differences in the *H. cunea* pupae from larvae reared on different host trees, free amino acids and their metabolites were investigated. The Liquid Chromatography-Tandem Mass Spectrometry (HPLC-MS/MS) method allows for the determination of a wide spectrum of up to 94 free amino acids and their metabolites (Table S1). Under our experimental conditions, 80 amino acids and their metabolites were identified and quantified in the Rps group, and 81 in each of the Mal and Asi groups, of which 80 were shared (Figure S1). The MRM parameters for each of free amino acids were provided in Table S3. The determined amino acid profiles consisted of proteinogenic amino acid (PAA) and non-proteinogenic amino acid (NPAA) present in varying amounts among the three groups (Table 1 and Table S4).

The total content of amino acid and their metabolites in the Mal group (31.57 ± 1.18 g/kg, wet mass, w.m.) was significantly lower than that in group Asi (35.11 ± 2.83 g/kg, w.m.), but showed no difference from the Rps group (32.96 ± 2.20 g/kg, w.m.). There was no significant difference among the three groups in total essential amino acid (EAA) content, while the total non-essential amino acid (NEAA) content in the Mal group was significantly lower than that in the other two groups (Table 1). The most abundant EAA were tryptophan (Trp) and lysine (Lys), and the most abundant NEAA were glutamine (Gln) and glutamic acid (Glu) (Table 1).

Trp content was the lowest in the Rps group (546.32 ± 68.61 mg/kg). The Mal group contained the lowest levels of Lys (348.84 ± 25.26 mg/kg). Gln content was present with lowest levels in the Mal group (2622.69 ± 167.21 mg/kg). There was no significant difference in the content of Glu among the three groups (Table 1). Similarly, there was no significant difference in the content of NPAA among the three groups. The content of NPAA accounted for over 62% of the total content, with Phosphorylethanolamine (PEA), Succinic-Acid (SA, 7607.20 ± 716.42 mg/kg–9973.60 ± 927.12 mg/kg) and argininosuccinic-acid (ASA, 3097.46 ± 725.73 mg/kg–4493.98 ± 434.95 mg/kg) being the most abundant components. PEA displayed the lowest levels in the Rps group (9325.82 ± 617.06 mg/kg), and highest in the Asi group (10,017.69 ± 476.70 mg/kg). SA and ASA exhibited the lowest levels in the Mal group (Table 1). Notably, γ-Aminobutyric-Acid (GABA) was detected, and there was no significant difference in the content of GABA among the three groups (Table 1).

**Figure 3:**
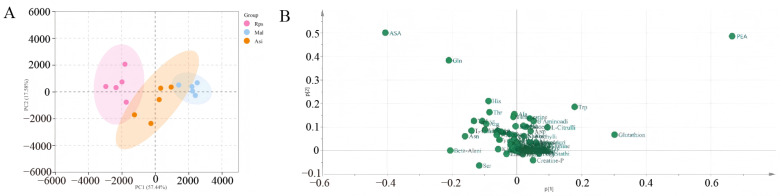
PCA model amino acid in *H. cunea* pupae. (**A**) Score plot of PCA model with R2X (cum); (**B**) Loading scatter plot of PCA model.

PCA was performed to examine variations in the free amino acid profiles of *H. cunea* pupae from the three groups. In the PCA score plot, pupae from the three host tree groups formed distinct clusters (Figure 3A). Notably, the Asi group exhibited greater intra-group variability in amino acid composition to the Rps and Mal groups. The loadings plot, which visualizes the original variables that contribute most to the sample grouping, revealed that ASA, Gln, and PEP had the strongest influence on the scaled data (Figure 3B).

### 3.4. Lipid Profiles of the H. cunea Pupae

#### 3.4.1. Lipid Profiles of the Three Dietary Groups

**Figure 4:**
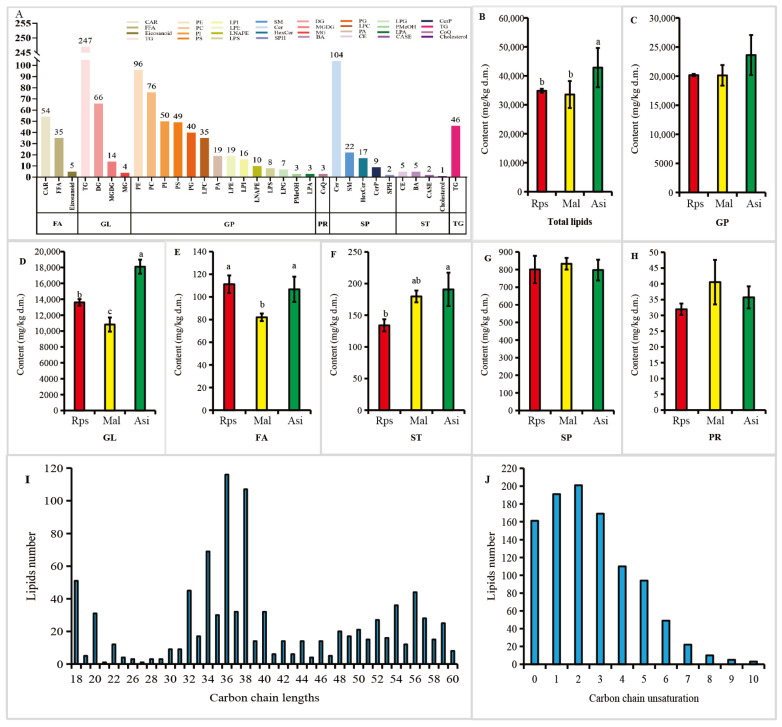
Lipid profiles of fall webworm pupae. (**A**) Lipid classes and subclasses. (**B**) Lipid contents. (**C**–**H**) GLs, GPs, FAs, STs, SPs, and PRs content. (**I**) Lipid carbon chanin lengths distribution histogram. (**J**) Lipid carbon chain unsaturation distribution histogram. The different lowercase letters indicated statistically significant differences at the 0.05 level. ANOVA followed by HSD post hoc test was used for multiple comparisons. Rps, Mal, and Asi represent the pupae of larvae reared on leaves of *R. pseudoacacia*, *M. alba* and *A. sibirica*, respectively.

The widely targeted lipidomic analysis was conducted using LC-ESI/MS/MS to profile lipids among the three groups of *H. cunea* pupae. Total ion chromatograms in positive and negative modes were shown in Table S5. A total of 1026 lipid molecules were identified and classified into six categories, including 431 glycerophospholipids (GPs), 331 glycerolipids (GLs), 154 sphingolipids (SPs), 94 fatty acyls (FAs), 13 sterol lipids (STs), and three prenol lipids (PRs), were identified in the fall webworm pupa (Figure 4A and Table S5). These lipids were further subdivided into 31 subclasses (Figure 4A and Table S6). Notably, the lipid profile exhibited considerable diversity, particularly within GLs, where TGs represented a major component. Unique lipid classes such as PRs were also present. From the perspective of major lipid classification, GPs were the primary lipid class and served as the main structural foundation of the lipid composition. Together, GPs, GLs and SPs dominated the overall lipid profile (Figure 4A).

Regarding total lipid content, the Asi group (42,852.73 ± 6782.18 mg/kg w.m.) contained significantly more than the Rps (34,849.96 ± 688.73 mg/kg, w.m.) and Mal (33,550.39 ± 4656.86 mg/kg, w.m.) groups (Figure 4B). GPs and GLs were the predominant classes. Although the distribution pattern of GPs mirrored the total lipid content, their absolute content did not differ significantly among groups (Figure 4C). In contrast, thecontent of GL was significantly higher in the Asi group than in the Rps and Mal groups (Figure 4D). FA content was significantly lower in the Mal group than in Rps and Asi groups. In terms of STs content, the Asi group possessed the highest amount (190.81 ± 52.87 mg/kg), but there was no significant difference from the other groups (Figure 4E). No significant differences were observed for SPs and PRs among groups, with PRs exhibiting the lowest average content across all lipid classes (Figure 4G,H).

Meanwhile, a systematic analysis was conducted on the carbon chain length and saturation of 1026 identified lipids. The results showed that lipid carbon chains ranged from 2 to 60 carbons in length. The carbon chain length distribution was similar across the three dietary groups, with long-chain lipids (≥14 carbons) predominating (>95%) and C36 being the most abundant in all groups (Figure 4I). Minor intergroup differences (e.g., a single lipid difference for chain lengths 8, 9, 10, 37, 39, 42 (Table S5). Regarding unsaturation, the number of double bonds per lipid molecule ranged from 0 to 10. The most common degrees of unsaturation were 2 (n = 201, 19.55%), 1 (n = 192, 18.68%), 3 (n = 169, 16.44%), and 0 (n = 162, 15.76%) (Figure 4J).

#### 3.4.2. Nutritional Quality Assessment

The nutritional quality of the *H. cunea* pupae was evaluated based on the free fatty acid, phospholipid and the presence of coenzymes.

**Figure 5:**
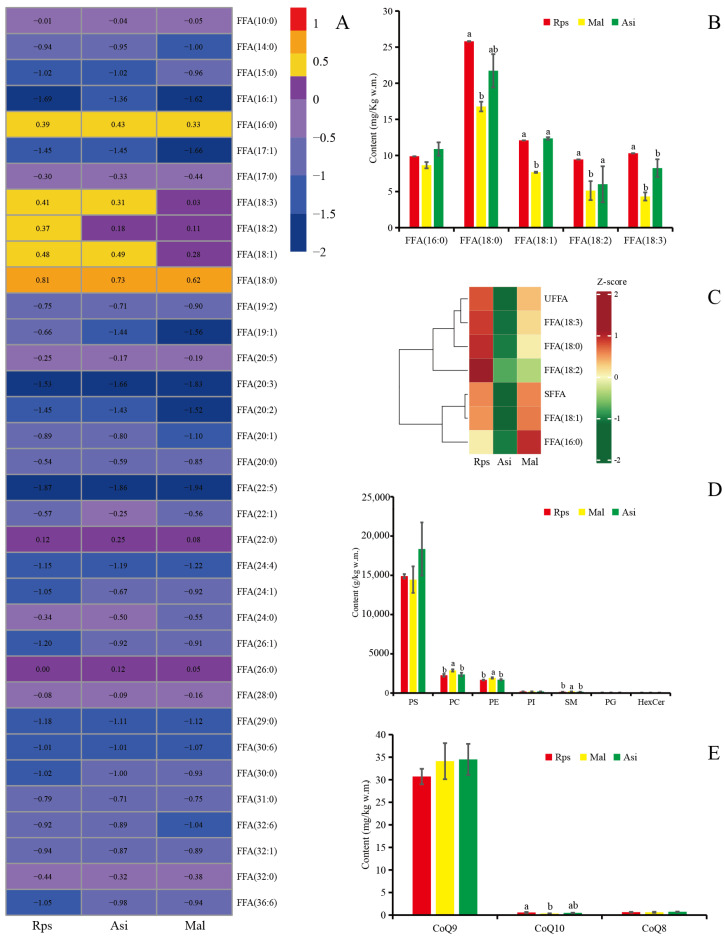
Free fatty acids and phospholipids in the fall webworm pupae. (**A**) Heatmap analysis of FFAs. (**B**) Comparison of the contents of C16:0, C18:0, C18:1, C18:2, and C18:3. (**C**) Heatmap analysis of the proportions of FFA(16:0), FFA(18:0), FFA(18:1), FFA(18:2), and FFA(18:3). (**D**) PL in the fall webworm pupa. (**E**) Comparison of the contents of CoQ8, CoQ9, and CoQ10. The different lowercase letters indicated statistically significant differences at the 0.05 level. ANOVA followed by HSD post hoc test was used for multiple comparisons. Rps, Mal, and Asi represent the pupae of larvae reared on leaves of *R. pseudoacacia*, *M. alba* and *A. sibirica*, respectively.

In the *H. cunea* pupae, 35 distinct free fatty acids (FFAs) were identified. The predominant FFAs were C16:0, C18:0, C18:1, C18:2, and C18:3, which collectively accounted for over 58% of the total FFA content (Figure 5A). Notably, the Rps group exhibited the highest content of C18:0 (25.80 ± 0.03 mg/kg), C18:2 (9.45 ± 0.01 mg/kg) and C18:3 (10.30 ± 0.01 mg/kg). In contrast, no significant difference was observed in the content of C18:1 and C16:0 among the three groups (Figure 5B). Cluster analysis via heatmap revealed that the Rps group showed elevated levels of C18 with zero, one, two, and three unsaturation levels. However, the Asi group displayed a higher level of C16:0. Furthermore, both the Rps and Asi groups contained significantly higher amounts of saturated free fatty acids (SFFAs), unsaturated free fatty acids (UFFAs), and the five major FFAs compared to the Mal group (Figure 5C). Additionally, the Rps group had the highest UFFA content, constituting 39.90% of total FFAs, followed by the Asi (38.09%) and Mal (34.21%) groups.

A total of 315 phospholipids, belonging to seven different types and containing up to eight unsaturated bonds, were identified across the three groups (Table S5). The most abundant type was phosphatidylserine (PS), followed by phosphatidylcholine (PC) and phosphatidylethanolamine (PE) (Figure 5D). Together, these three phospholipids accounted for over >97% of the total content of phospholipid. Although the Asi group showed the highest mean level of PS (18,346.87 ± 6783.06 mg/kg), the differences compared to the Rps (14,884.31 ± 542.30 mg/kg) and Mal (14,447.04 ± 3376.04 mg/kg) groups were not statistically significant. In contrast, the contents of both PC and PE in the Mal group were significantly higher than those in the Rps and Asi groups (Figure 5D).

Three types of coenzyme were detected in the *H. cunea* pupae, namely CoQ8, CoQ9, and CoQ10, with CoQ9 constituting more than 96% of the total. The CoQ9 content was the highest in Asi group (34.50 ± 3.45 mg/kg), but there was no significant difference from the other two groups (Figure 5E).

#### 3.4.3. Chemometrics-Based Discrimination of Lipidomic Signatures

**Figure 6:**
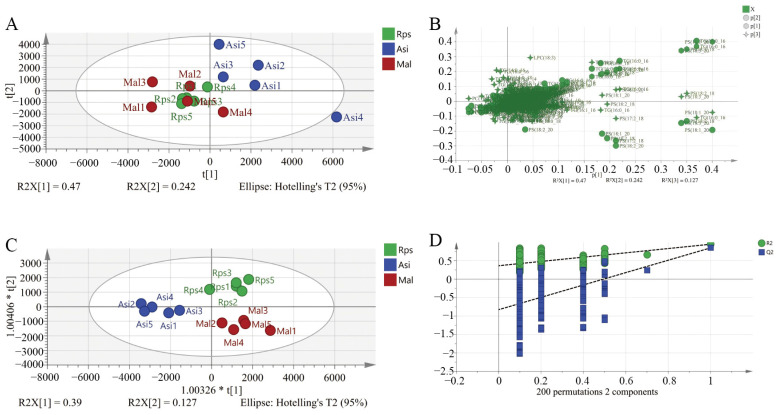
PCA and OPLS-DA model of lipids in *H. cunea* pupae. (**A**) Score plot of PCA model; (**B**) loading scatter plot of PCA model; (**C**) score plot of OPLS-DA model; (**D**) permutation tests of the OPLSD-DA model.

To comprehensively compare the lipidomic profiles and identify differentially abundant lipids among the three groups, systematic multivariate statistical analyses were conducted. PCA was first performed to examine the overall variation. The PCA plot showed that samples from the three groups of pupae did not form three distinct, well-separated clusters, and both intra-group cohesion and inter-group distinctness were limited (Figure 6A). The model parameters were *R*^2^*X*(cum) value of 0.812 and *Q*^2^(cum) value of 0.585. The corresponding loading scatter plot (Figure 6B) revealed variable correlations, with closely positioned variables indicating positive correlation and distantly opposed ones suggesting negative correlation, consistent with the PCA result.

Given the insufficient group separation in the unsupervised PCA model, a supervised OPLS-DA was employed to maximize the separation among the predefined groups. In the OPLS-DA score plot, the samples were clearly clustered into three groups along the first predictive component, with an *R*^2^*X*(cum) of 0.835, an *R*^2^*Y*(cum) of 0.91 and a *Q*^2^(cum) of 0.811 (Figure 6C). These values indicate good explanatory power and predictive ability of the model. The robustness and validity of the OPLS-DA model were further assessed by a permutation test (n = 200). The regression intercepts of R^2^ and Q^2^ were 0.376 and -0.809, respectively, confirming that the model was not overfitted and was statistically reliable for identifying differential lipids (Figure 6D).

#### 3.4.4. The Differential Lipids Among the Three Groups

Based on the OPLS-DA results, significantly differential lipids were identified using the criteria of VIP > 1 and *p*-value < 0.05. A total of 264 differential lipids were identified among the three groups (Table S7), which were distributed across 6 major lipid classes (GPs, n = 119; GLs, n = 94; FA, n = 29; TGs, n = 12; SPs, n = 6; ST, n = 4).

**Figure 7:**
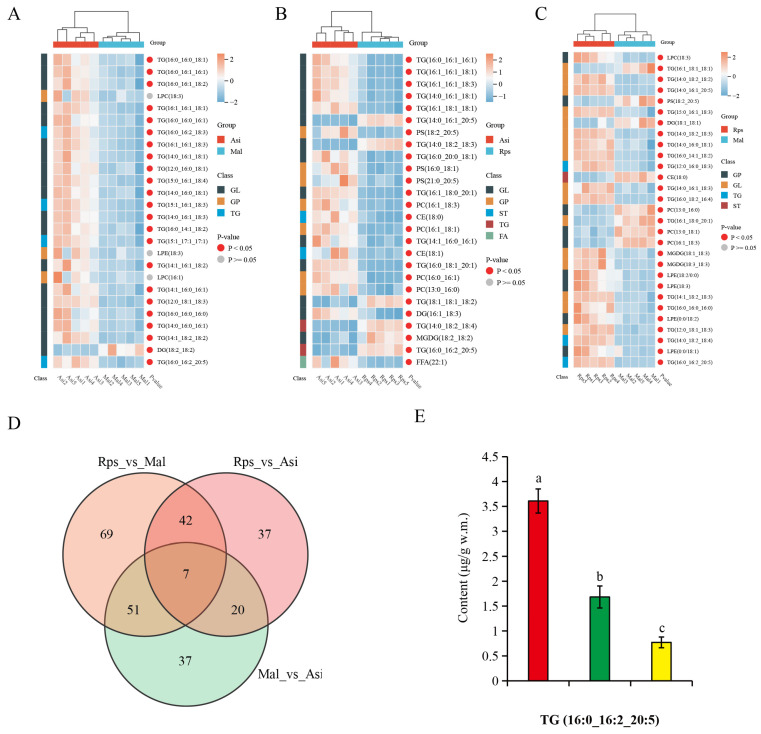
Differential lipid analysis of *H. cunea* pupae. (**A**–**C**) The combined heatmap of differential lipids with higher content in Asi vs. Mal comparison, Asi vs. Rps comparison, and Rps vs. Mal comparison; (**D**) Venn diagram showing the numbers of significantly differential lipids identified in each pairwise comparison; (**E**) bar plot of TG (16:0_16:2_20:5), the different lowercase letters indicated statistically significant differences at the 0.05 level, ANOVA followed by HSD post hoc test was used for multiple comparisons. Rps, Mal, and Asi represent the pupae of larvae reared on leaves of *R. pseudoacacia*, *M. alba* and *A. sibirica*, respectively.

In the Mal_vs_Asi comparison, a total of 114 lipids with significant differences in abundance were identified (VIP > 1, *p* < 0.05, FC > 2.0 or <0.5), of which 96 were significantly increased and 19 were decreased (Table S8). Lipidomic analysis revealed that the predominant differentially expressed lipids belonged to GLs, GPs and TGs. Among these, GLs were significantly elevated in the Asi group. Specifically, 22 TGs showed a marked increase, particularly TG (16:0_16:0_18:1) (1321.57 ± 330.76 mg/kg, 2.33-fold increase) and TG (16:0_16:1_16:1) (841.70 ± 240.38 mg/kg, 4.09-fold increase), indicating substantial accumulation in the Asi group relative to the Mal group. The Asi group also exhibited increased levels of 3 GPs [LPC (18:3), LPC (16:1), LPE (18:3)] compared to the Mal group. Conversely, DG (18:2_18:2) was significantly downregulated in Asi group (Figure 7A).

A total of 106 lipids with significant variation in abundance were identified in the Rps_vs_Asi comparison (VIP > 1, *p* < 0.05, FC > 2.0 or <0.5), including 77 with significantly increased abundance and 29 with decreased abundance (Table S9). The differential lipid profile was similarly dominated by GLs and GPs. While the levels of GLs remained significantly elevated in the Asi group, particularly TG (16:0_16:1_16:1) (5.01-fold increase), and TG (16:1_16:1_18:1) (357.66± 63.53 mg/kg, 3.41-fold increase), TG (16:1_16:1_18:3) (3.39-fold increase), and TG (14:0_16:1_18:1) (3.14-fold increase). However, the Rps group exhibited marked increases in six GLs [TG (14:0_16:1_20:5), TG (14:0_18:2_18:3), TG (18:1_18:1_18:2), TG (14:0_18:2_18:4), TG (16:0_16:2_20:5), and MGDG (18:2_18:2)]. It was noteworthy that two STs and one FA, CE (18:0), CE (18:1) and FFA (22:1), were also significantly elevated in the Asi group (Figure 7B).

In the Rps_vs_Mal comparison, 169 differential lipids were identified (51 upregulated, 118 downregulated), predominantly comprising GPs and GLs (Table S10). GPs showed remarkable accumulation in the Rps group, comprising LPC (18:3) (481.77 ± 238.36 mg/kg, 3.18-fold), LPE (18:3) (41.76 ± 16.37 mg/kg, 4.20-fold) and LPE (18:2/0:0) (39.38 ± 14.67 mg/kg, 3.80-fold). GLs, particularly TG (14:0_16:1_20:5) (215.48 ± 14.47 mg/kg, 3.63-fold), TG (14:0_18:2_18:2) (178.10 ± 25.94 mg/kg, 2.33-fold) and TG (16:0_14:1_18:2) (108.07 ± 6.36 mg/kg, 3.52-fold), were significantly elevated in the Rps group compared to the Mal group. Conversely, three GLs [TG (16:1_18:1_18:1), TG (16:1_18:0_20:1), and DG (18:1_18:1)] and four GPs [PS (18:2_20:5), PC (13:0_16:0), PC (13:0_16:0), PC (13:0_18:1), and PC (16:1)18:3)] and CE (18:0) showed higher abundance in the Mal group (Figure 7C).

The Venn diagram revealed seven common lipids that were differentially expressed across all three comparisons (Figure 7D). Notably, TG (16:0_16:2_20:5) demonstrated the highest content in Rps, and its level was significantly higher than those in the Asi and Mal groups (Figure 7E), establishing it as a distinctive lipid marker among the three groups.

## 4. Discussion

The fall webworm is a globally significant quarantine pest, known to feed on over 600 plant species [13]. Due to its highly polyphagous nature and strong adaptability, *H. cunea* has infested a cumulative area of over 7.3 × 10^5^ hm^2^ as of 2022, since its first detection in China in 1979 [20]. Previous research on *H. cunea* has primarily focused on quarantine measures and control strategies. This study analyzes the nutritional composition of its pupae to assess their potential for resource utilization. Furthermore, by analyzing pupae obtained from larvae reared on three different host plants, this study also explores how host plant variation influences the nutritional plasticity of *H. cunea*, thereby shedding light on the physiological basis of its adaptability to diverse environments.

In this study, the morphological traits and proximate composition of *H. cunea* pupae were evaluated. Pupae from the Rps and Mal groups showed significantly greater body weight, length and width compared to those Asi group, consistent with previously reported larval feeding rates [16]. Body size is a key fitness-related trait in insects, as it positively correlates with fecundity, longevity, stress resistance and mating success [21]. In contrast, no significant differences were observed in dry matter, crude protein and crude fat content among the three groups. Insects are enriched in protein and fat, with reported ranges of 8.8–80% and 7.9–40%, respectively [22,23]. The crude protein content of *H. cunea* pupae was notably high, averaging 64.94 ± 1.57% (d.m.), and was not influenced by dietary source. Similarly, the average crude fat content was 30.82 ± 0.53% (d.m.), with no significant variation across diets. This value falls within the upper range of crude fat contents reported for *T. molitor* larvae (19.1–43.08% d.m.), a well-recognized insect species for food and feed applications [24]. Furthermore, this study confirms that *H. cunea* pupae contained significantly higher levels of Mg and Ca. Discrepancies between declined pupal size and stable nutritional composition indicate that host plant quality restricts the somatic growth of *H. cunea* instead of nutrient assimilation and storage. This finding aligns with the resource allocation priority hypothesis, whereby nutritionally stressed insects sustain internal nutrient reserves for post-emergence fitness by sacrificing body growth [25].

Free amino acids play a crucial role in influencing the physiology and metabolism of animals. In this study, 82 free amino acids and their metabolites were identified and quantified in the *H. cunea* pupae using UPLC and ESI-MS/MS methods. Glutamine, glutamic acid and tryptophan were the most abundant amino acids, while L-cystine was present at the lowest concentration. All the ten essential amino acids were detected, underscoring the nutritional value of the pupae. Host plant variation has been shown to significantly affect the development, digestive physiology, and metabolic adaptation of *H. cunea* larvae [26,27]. Although larvae fed on *A. sibirica* (Asi group) exhibited the lowest feeding rate [16], this group showed the highest total free amino acid and their metabolites content among the three groups. Tryptophan levels were highest in the Mal group, though the difference between the Asi and Mal groups was not statistically significant. Interestingly, this paradoxical pattern suggests a compensatory metabolic response. Reduced food intake may trigger enhanced amino acid accumulation or reduced catabolism during metamorphosis, thereby allowing the insect to maintain amino acid reserves under suboptimal nutritional conditions [28]. The variation in tryptophan and other EAA levels across host groups further supports the existence of host-plant-driven metabolic plasticity in *H. cunea*, which may contribute to its dietary adaptability and invasion success.

PEA and SA were the predominant NPAA in *H. cunea* pupae, with their contents varying across the three sample groups. PEA, a precursor of the membrane lipid phosphatidylethanolamine, is one of the main phosphate compounds in lepidopterous insects [29,30]. Variation in PEA levels across host plant groups may reflect differences in membrane synthesis and turnover, which could be linked to metabolic adjustments in response to host plant quality [31]. SA enhances tissue oxygenation, stabilizes mitochondria structure and function, and helps regulate cellular ion metabolism [32]. The detection of SA in *H. cunea* pupae, and its variation among host groups, suggests that host-plant-driven metabolic shifts may affect energy metabolism and stress responses, which are central to dietary adaptability. Interestingly, gamma-aminobutyric acid (GABA) was also detected in *H. cunea* pupae. GABA is a naturally occurring potential bioactive compound present in plants, microorganisms, animals, and humans [33]. In insects, GABA is also involved in stress tolerance and metabolic regulation under suboptimal conditions. The consistent presence of GABA across different host plants suggests a constitutive role in maintaining physiological homeostasis, which may contribute to the pest’s metabolic plasticity and ability to cope with diverse nutritional environments [34].

We performed a comprehensive lipidomic analysis of the fall webworm pupae from the larvae fed on different host trees. Previous reports have indicated that TGs make up the majority of insect lipids [21]. In this study, 1026 lipids were identified in the *H. cunea* pupae, which were classified into six major categories, with TGs representing the predominant lipid subclasses. The structural and functional properties of lipids, including their digestibility, are influenced by both chain length and degree of unsaturation [35]. Lipids in *H. cunea* pupae were characterized by a high proportion of long-chain lipid species (up to 96.11%) and unsaturated lipid species (up to 98.74%). Given the documented benefits of long-chain and unsaturated fatty acids on animal growth performance, immune health, and product quality, these findings highlight the potential of *H. cunea* pupae as a functional lipid source for animal feed application. This lipid profile also reflects the metabolic plasticity of *H. cunea*, which facilitates its dietary adaptability and invasion success [36].

The lipid composition and content were determined by the host plant. These host-driven differences in lipid profiles are consistent with the species’ ability to adjust its digestive and metabolic physiology to different host plants, as demonstrated by alterations in larval development, survival, and enzyme activities [26]. Although the larvae of *H. cunea* showed the lowest feeding rate on *A. sibirica*, the Asi group exhibited the highest total lipid content. The results of this study indicate that when developing on suboptimal host plants, *H. cunea* adopts a metabolic compensation strategy to allocate more resources to lipid accumulation under nutritional stress. This finding is consistent with previous research by Sellers et al., which reported an increase in lipid supply in female moth eggs of *H. cunea* raised on low-quality host plants [37]. In addition, the oils extracted from *H. cunea* pupae were rich in PS, PC and PE, which were the main components of lecithin [38]. Lecithin functions as a multifunctional surfactant in food products, serving as an antioxidant, flavor protector, and emulsifier [39].

A total of 35 FFAs were identified in *H. cunea* pupae. The content of SFFA exceeded that of UFFA. The predominant individual FFAs were C18:3 (α-linolenic acid) and C18:2 (linoleic acid). Notably, both C18:3 and C18:2 reached their highest concentrations in the Rps group compared with the other groups. As precursors to signaling molecules involved in regulating metabolism, inflammation, and reproduction, linoleic acid and α-linolenic acid play crucial physiological roles [40,41]. The variation in their concentrations across host plant groups suggests that the fatty acid composition of *H. cunea* pupae is influenced by host plant quality, which may reflect the metabolic plasticity that facilitates the pest’s dietary adaptability [42].

Additionally, *H. cunea* pupae contained substantial levels of coenzyme Q9. Coenzyme Q is an essential component of the mitochondrial oxidative phosphorylation system, where it functions as a lipid-soluble electron carrier for cellular energy production [43]. In mammals, the protein encoded by the CoQ9 gene plays a critical role in coenzyme Q biosynthesis. Clinical studies have shown that mutations in the CoQ9 gene can lead to autosomal-recessive neonatal-onset primary coenzyme Q10 deficiency, a severe and treatable form of mitochondrial disease [44]. However, our study only detected the presence of CoQ9 in the pupae. No bioavailability, toxicological, or feeding trials were conducted. Therefore, the detection of CoQ9 in *H. cunea* pupae suggests that further investigation into its potential bioactivity is warranted. The levels of CoQ9 did not differ significantly among the three host plant groups. This constitutive presence suggests that CoQ9 may serve as a fundamental metabolic component that maintains basal mitochondrial function across different nutritional environments [45], thereby contributing to the metabolic stability of *H. cunea* as it adapts to diverse host plants.

Pupae are immobile, easy to collect, and nutritionally denser than larvae, although we did not assess the feasibility or biosecurity risks of large-scale production. The observed host-driven variation in pupal lipids and amino acids is consistent with previous findings [20,26], but direct testing in *H. cunea* is needed. Regarding safety, larval setae are largely shed during pupation, and many lepidopteran pupae with similar larval hairs have been traditionally consumed [46,47], suggesting that *H. cunea* pupae could be safe after proper processing. However, the present study did not analyze digestibility, bioavailability, anti-nutritional factors, or comprehensive safety endpoints; these must be addressed before any practical application.

## 5. Conclusions

We conclude that *H. cunea* pupae contain a complete profile of essential and non-essential amino acids, as well as diverse lipids. Host-plant-driven variations in these nutrient profiles reflect the metabolic plasticity and dietary adaptability of this polyphagous pest, which are key traits underlying its invasion success. These findings also suggest a potential use of *H. cunea* pupae as a nutrient-rich ingredient for feed, pending safety assessments.

## Figures and Tables

**Table 1:** Contents of PAA, NPAA, and the ten most abundant amino-acid-associated metabolites in *H. cunea* pupae.

Compounds	Mean Content (mg/kg, w.m.)		
	Rps	Mal	Asi
proteinogenic amino acid (PAA)			
Tryptophan ^E^	546.32 ± 68.61 ^b^	831.92 ± 92.95 ^a^	763.73 ± 113.82 ^a^
Lysine ^E^	463.23 ± 37.89 ^a^	348.84 ± 25.26 ^b^	424.29 ± 68.93 ^a^
Histidine ^E^	448.50 ± 69.88 ^a^	330.14 ± 12.48 ^b^	443.07 ± 108.30 ^a^
Threonine ^E^	311.32 ± 55.53 ^a^	226.90 ± 22.55 ^b^	347.76 ± 79.19 ^a^
Arginine ^E^	284.60 ± 35.54 ^a^	197.41 ± 20.53 ^b^	260.63 ± 45.16 ^a^
Valine ^E^	157.39 ± 8.02 ^a^	120.52 ± 7.40 ^b^	159.72 ± 23.76 ^a^
Isoleucine ^E^	135.76 ± 12.44 ^a^	102.61 ± 3.26 ^b^	130.24 ± 19.47 ^a^
Leucine ^E^	124.45 ± 8.83	102.37 ± 7.06	116.94 ± 24.98
Methionine ^E^	106.26 ± 15.70	116.19 ± 22.24	122.04 ± 21.77
Phenylalanine ^E^	54.58 ± 6.30	45.26 ± 1.12	58.28 ± 13.01
Total EAA	2914.23 ± 257.00	2705.92 ± 104.52	3163.77 ± 494.73
Glutamine ^NE^	3150.71 ± 235.96 ^a^	2622.69 ± 167.21 ^b^	3128.11 ± 402.28 ^a^
Glutamic acid ^NE^	2289.64 ± 188.80	2377.07 ± 205.08	2344.09 ± 253.93
Alanine ^NE^	281.82 ± 21.10	283.76 ± 23.18	337.06 ± 67.62
Aspartic acid ^NE^	255.79 ± 35.00 ^a^	76.19 ± 14.79 ^c^	185.19 ± 49.25 ^b^
Tyrosine ^NE^	238.48 ± 18.11 ^a^	95.10 ± 21.88 ^b^	230.76 ± 59.11 ^a^
Serine ^NE^	241.94 ± 50.88 ^a^	146.28 ± 9.07 ^b^	170.73 ± 43.69 ^b^
Glycine ^NE^	210.76 ± 16.43 ^ab^	178.66 ± 28.17 ^b^	226.03 ± 31.60 ^a^
Proline ^NE^	137.40 ± 13.81 ^b^	150.27 ± 12.00 ^ab^	166.45 ± 12.18 ^a^
Aspartate ^NE^	76.03 ± 13.62	107.47 ± 22.28	98.57 ± 29.38
L-Cystine ^NE^	0.68 ± 0.42 ^b^	6.36 ± 2.81 ^a^	3.77 ± 1.25 ^a^
Total NEAA	6601.42 ± 296.71 ^a^	5760.10 ± 350.70 ^b^	6553.71 ± 745.67 ^a^
Total PAA	9515.65 ± 486.19 ^ab^	8466.03 ± 394.44 ^b^	9717.48 ± 1219.55 ^a^
non-proteinogenic amino acid (NPAA)			
Phosphorylethanolamine	6952.77 ± 958.54 ^b^	10017.69 ± 476.70 ^a^	9325.82 ± 617.06 ^a^
Succinic-Acid	9973.60 ± 927.12 ^a^	7607.20 ± 716.42 ^b^	9158.38 ± 926.53 ^a^
argininosuccinic-acid	4493.98 ± 434.95 ^a^	3097.46 ± 725.73 ^b^	3981.32 ± 957.11 ^ab^
β-Alanine	401.77 ± 182.57 ^a^	87.04 ± 24.44 ^b^	260.84 ± 128.27 ^ab^
Urea	273.56 ± 22.19 ^a^	168.59 ± 20.67 ^b^	260.28 ± 34.37 ^a^
L-Ornithine	261.07 ± 36.10 ^a^	97.14 ± 19.25 ^b^	257.73 ± 80.77 ^a^
Methionine-Sulfoxide	158.67 ± 41.72	201.25 ± 38.36	186.88 ± 23.20
1-Methylhistidine	135.19 ± 9.95 ^a^	100.34 ± 9.42 ^a^	131.09 ± 13.83 ^b^
γ-Aminobutyric-Acid	111.36 ± 15.20	77.61 ± 34.68	110.81 ± 18.79
α-Aminoadipic-acid	97.72 ± 43.15 ^b^	126.19 ± 20.98 ^b^	192.19 ± 47.21 ^a^
Total NPAA (g/kg)	23.44 ± 1.89	23.11 ± 0.84	25.39 ± 1.74
Total (g/kg)	32.96 ± 2.20 ^ab^	31.57 ± 1.18 ^b^	35.11 ± 2.83 ^a^

Note: Values are means ± SD (n = 5), followed by different superscript letters are significantly different (*p* ≤ 0.05). ANOVA followed by HSD post hoc test was used for multiple comparisons. Rps, Mal, and Asi represent the pupae of larvae reared on leaves of *R. pseudoacacia*, *M. alba* and *A. sibirica*, respectively. ^E^, essential amino acid; ^NE^, non-essential amino acid.

## Data Availability

The original contributions presented in this study are included in the article/Supplementary Materials. Further inquiries can be directed to the corresponding author.

## Supplementary Materials

The following supporting information can be downloaded at: https://www.mdpi.com/article/10.3390/insects17060590/s1, Figure S1. Veen plot of amino acids and their metabolites detected in *H. cunea* pupae. Table S1. Amino acids and their derivatives detected in this study and their standard curves. Table S2. The lipid internal standards. Table S3. MRM parameters for the 82 free amino acids detected in the *H. cunea* pupae. Table S4. Amino acid and associated metabolite content in the *H. cunea* pupae. Table S5. Lipid contents (nmol/g, w.m.) of *H. cunea* pupae. Table S6. Full name and abbreviation of secondary classification of lipid. Table S7. Differential lipids of *H. cunea* pupae (mg/kg w.m.). Table S8. The significantly differential lipids in the Mal_vs_Asi comparison (mg/kg w.m.). Table S9. The significantly differential lipids in the Rps_vs_Asi comparison (mg/kg w.m.). Table S10. The significantly differential lipids in the Rps_vs_Mal comparison (mg/kg w.m.).
