# Supplementary material for: Association of Different Host Diets with the Nutritional Composition of the Fall Webworm, Hyphantria cunea Drury (Lepidoptera: Erebidae)
Source: Insects. 2026 Jun 4;17(6):590. doi: 10.3390/insects17060590 (PMC13300641; doi:10.3390/insects17060590)
Supplement: Supplementary file 1 [file insects-17-00590-s001.zip › Table S/Table S4.pdf]

Table S4. Amino acid and associated metabolite content in the *H. cunea* pupae reared on different host trees.

**Compounds**	**Mean content (mg/kg, w.m.)**		
	**Rps**	**Mal**	**Asi**
proteinogenic amino acid (PAA)			
**Tryptophan**^{E}	546.32±68.61^{b}	831.92±92.95^{a}	763.73±113.82^{a}
**Lysine**^{E}	463.23±37.89^{a}	348.84±25.26^{b}	424.29±68.93^{a}
**Histidine**^{E}	448.50±69.88^{a}	330.14±12.48^{b}	443.07±108.30^{a}
**Threonine**^{E}	311.32±55.53^{a}	226.90±22.55^{b}	347.76±79.19^{a}
**Arginine**^{E}	284.60±35.54^{a}	197.41±20.53^{b}	260.63±45.16^{a}
**Valine**^{E}	157.39±8.02^{a}	120.52±7.40^{b}	159.72±23.76^{a}
**Isoleucine**^{E}	135.76±12.44^{a}	102.61±3.26^{b}	130.24±19.47^{a}
**Leucine**^{E}	124.45±8.83	102.37±7.06	116.94±24.98
**Methionine**^{E}	106.26±15.70	116.19±22.24	122.04±21.77
**Phenylalanine**^{E}	54.58±6.30	45.26±1.12	58.28±13.01
Total EAA	2914.23±257.00	2705.92±104.52	3163.77±494.73
**Glutamine**^{NE}	3150.71±235.96^{a}	2622.69±167.21^{b}	3128.11±402.28^{a}
**Glutamic acid**^{NE}	2289.64±188.80	2377.07±205.08	2344.09±253.93
**Alanine**^{NE}	281.82±21.10	283.76±23.18	337.06±67.62
**Aspartic acid**^{NE}	255.79±35.00^{a}	76.19±14.79^{c}	185.19±49.25^{b}
**Tyrosine**^{NE}	238.48±18.11^{a}	95.10±21.88^{b}	230.76±59.11^{a}
**Serine**^{NE}	241.94±50.88^{a}	146.28±9.07^{b}	170.73±43.69^{b}
**Glycine**^{NE}	210.76±16.43^{ab}	178.66±28.17^{b}	226.03±31.60^{a}
**Proline**^{NE}	137.40±13.81^{b}	150.27±12.00^{ab}	166.45±12.18^{a}
**Aspartate**^{NE}	76.03±13.62	107.47±22.28	98.57±29.38
**L-Cystine**^{NE}	0.68±0.42^{b}	6.36±2.81^{a}	3.77±1.25^{a}
Total NEAA	6601.42±296.71^{a}	5760.10±350.70^{b}	6553.71±745.67^{a}
Total PAA	9515.65±486.19^{ab}	8466.03±394.44^{b}	9717.48±1219.55^{a}
non-proteinogenic amino acid (NPAA)			
Phosphorylethanolamine	6952.77±958.54^{b}	10017.69±476.70^{a}	9325.82±617.06^{a}
Succinic-Acid	9973.60±927.12^{a}	7607.20±716.42^{b}	9158.38±926.53^{a}
argininosuccinic-acid	4493.98±434.95^{a}	3097.46±725.73^{b}	3981.32±957.11^{ab}
β-Alanine	401.77±182.57^{a}	87.04±24.44^{b}	260.84±128.27^{ab}
Urea	273.56±22.19^{a}	168.59±20.67^{b}	260.28±34.37^{a}
L-Ornithine	261.07±36.10^{a}	97.14±19.25^{b}	257.73±80.77^{a}
Methionine-Sulfoxide	158.67±41.72	201.25±38.36	186.88±23.20
1-Methylhistidine	135.19±9.95^{a}	100.34±9.42^{a}	131.09±13.83^{b}
**γ-Aminobutyric-Acid**	111.36±15.20	77.61±34.68	110.81±18.79
α-Aminoadipic-acid	97.72±43.15^{b}	126.19±20.98^{b}	192.19±47.21^{a}
Glutathione-Oxidized	96.32±46.24^{b}	876.30±381.60^{a}	702.34±278.75^{a}
2-Aminoethanesulfonic-Acid	83.95±18.38	76.93±12.49	98.17±13.12
Homoserine	77.08±20.32^{b}	61.93±6.77^{b}	124.46±29.37^{a}
Nicotinuric-Acid	66.89±14.27^{b}	95.92±10.22^{a}	80.21±8.19^{b}
O-Phospho-L-Serine	27.23±4.77	28.13±3.81	26.41±2.26
Kinurenine	24.80±3.51^{a}	3.21±1.81^{c}	12.23±4.96^{b}
L-Citrulline	23.61±39.25^{b}	126.88±82.26^{ab}	189.77±76.73^{a}
Homo-Arg	23.72±3.45^{b}	29.56±2.11^{a}	26.07±5.51^{ab}
N'-Formylkynurenine	15.77±7.77^{b}	21.40±2.16^{b}	32.01±3.07^{a}
Trans-4-Hydroxy-L-Proline	12.41±3.81^{b}	19.02±1.27^{a}	15.90±2.46^{ab}
(5-L-Glutamyl)-L-Amino-Acid	12.34±3.30^{b}	6.83±0.68^{c}	17.84±5.77^{a}

Ethanolamine	13.15±2.40[a]	5.22±4.49[b]	7.42±2.09[b]
5-Hydroxylysine	10.43±1.38[a]	5.66±0.38[b]	10.36±1.56[a]
L-Cystathionine	10.80±3.73[c]	42.62±2.88[a]	26.11±5.78[b]
γ-Glutamate-Cysteine	9.92±6.85	9.42±5.10	38.56±33.24
TRP-GLU	9.46±2.68[ab]	5.57±1.68[b]	13.79±9.06[a]
3-Aminoisobutanoic-Acid	8.29±1.88[ab]	6.73±1.58[b]	10.88±4.10[a]
L-Pipecolic-Acid	6.91±2.37[b]	25.69±14.66[a]	29.85±10.21[a]
Nα-Acetyl-L-Arginine	6.59±2.51[a]	3.96±0.94[b]	4.43±0.41[b]
(S)-β-Aminoisobutyric-Acid	7.10±1.51[b]	6.24±0.80[b]	9.90±2.17[a]
L-Carnosine	4.04±1.69	1.74±0.18	3.60±1.36
3-N-Methyl-L-Histidine	3.56±0.58[b]	4.21±0.62[ab]	5.69±1.92[a]
Glycyl-L-Proline	2.98±0.20[a]	1.72±0.42[b]	2.95±0.54[a]
N-Glycyl-L-Leucine	3.54±0.58	2.44±1.80	4.48±1.79
5-Hydroxy-tryptophan	1.59±0.46	1.00±0.29	1.71±0.82
3-Chloro-L-Tyrosine	0.56±0.10[a]	0.28±0.03[b]	0.50±0.11[a]
glycylphenylalanine	0.74±0.19	0.55±0.18	0.74±0.13
Trimethylamine-N-Oxide	0.05±0.02[b]	0.50±0.20[a]	0.04±0.02[b]
S-(5-Adenosyl)-L-Homocysteine	1.85±0.43[b]	3.09±0.48[b]	2.88±0.66[a]
2-Aminobutyric-acid	1.78±0.21	1.41±0.30	1.98±0.61
3-Hydroxyhippuric-Acid	1.56±0.31[b]	3.08±0.45[a]	1.76±0.28[b]
N6-Acetyl-L-Lysine	1.43±0.49[b]	2.19±0.63[a]	2.42±0.52[a]
4-Acetamidobutyric-Acid	1.23±0.08[b]	1.04±0.42[b]	1.68±0.30[a]
5-Aminovaleric-Acid	1.29±0.26[b]	3.11±0.86[a]	1.81±0.77[b]
N8-Acetylspermidine	1.19±0.14	0.90±0.11	1.31±0.51
N-Acetylaspartate	1.11±0.47[b]	2.07±0.56[ab]	2.81±1.13[a]
L-Homocitrulline	1.12±0.08[a]	0.83±0.08[b]	1.15±0.16[a]
Sarcosine	0.90±0.14	1.58±0.77	0.98±0.24
N-Isovaleroylglycine	0.88±0.07	1.17±0.33	1.08±0.22
N-Acetyl-L-Tyrosine	0.53±0.07[a]	0.30±0.02[c]	0.41±0.08[b]
D-Alanyl-D-Alanine	0.43±0.13[b]	1.00±0.26[ab]	1.89±1.12[a]
P-Aminohippuric-Acid	0.45±0.10[a]	0.29±0.02[b]	0.39±0.10[ab]
Creatine	0.33±0.01[b]	0.34±0.01[a]	0.33±0.01[ab]
3-Iodo-L-Tyrosine	0.28±0.00[ab]	0.27±0.01[b]	0.29±0.02[a]
N-Propionylglycine	0.29±0.02	0.41±0.27	0.30±0.04
Asp-Phe	0.20±0.01	0.19±0.03	0.21±0.02
Kynurenic-Acid	0.16±0.06[b]	1.03±0.39[a]	0.24±0.08[b]
S-Sulfo-L-Cysteine	0.04±0.01[b]	NA	0.42±0.13[a]
1,3,7-Trimethyluric-Acid	0.03±0.01[a]	0.01±0.00[b]	0.02±0.00[a]
L-tyrosine-methyl-ester	0.01±0.00[a]	0.003±0.00[b]	0.01±0.00[a]
Creatine-Phosphate	NA	34.63±26.02	NA
L-Theanine	NA	0.42±0.00[b]	0.43±0.01[a]
Total NPAA (g/kg)	23.44±1.89	23.11±0.84	25.39±1.74
Total (g/kg)	32.96±2.20[ab]	31.57±1.18[b]	35.11±2.83[a]
