# Supplementary figures and images for: Association of Different Host Diets with the Nutritional Composition of the Fall Webworm, Hyphantria cunea Drury (Lepidoptera: Erebidae)
Source: Insects. 2026 Jun 4;17(6):590. doi: 10.3390/insects17060590 (PMC13300641; doi:10.3390/insects17060590)

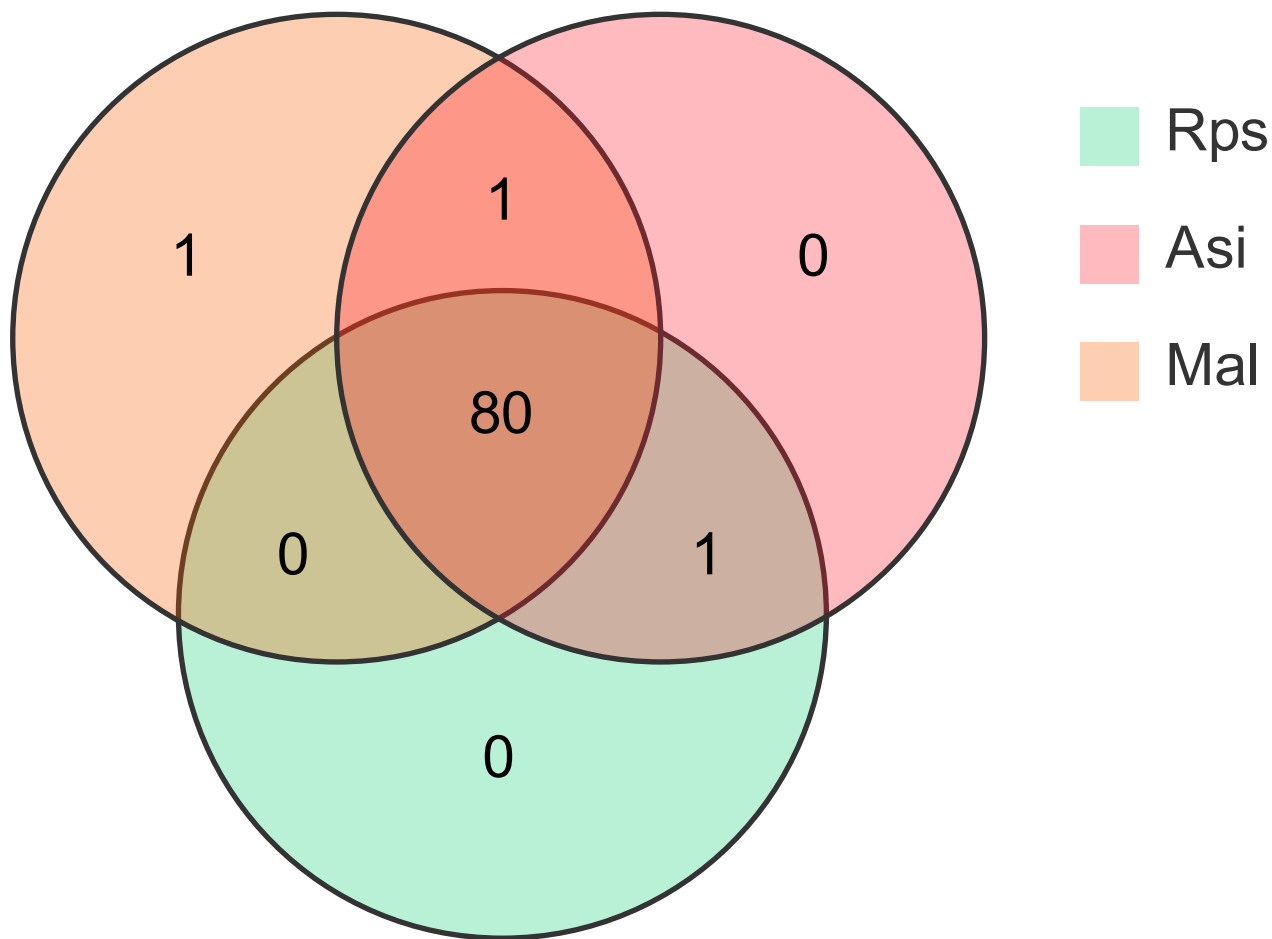

Supplement: Supplementary file 1 [file insects-17-00590-s001.zip › Figure S1.pdf]
